# Mercury Toxicity and Treatment: A Review of the Literature

**DOI:** 10.1155/2012/460508

**Published:** 2011-12-22

**Authors:** Robin A. Bernhoft

**Affiliations:** ^1^Bernhoft Center for Advanced Medicine, Suite 208, 11677 San Vicente Boulevard, Los Angeles, CA 90049, USA; ^2^Los Angeles Center for Advanced Medicine, Brentwood Gardens Suite 208, 11677 San Vicente Boulevard, Los Angeles, CA 90049, USA

## Abstract

Mercury is a toxic heavy metal which is widely dispersed in nature. Most human exposure results from fish consumption or dental amalgam. Mercury occurs in several chemical forms, with complex pharmacokinetics. Mercury is capable of inducing a wide range of clinical presentations. Diagnosis of mercury toxicity can be challenging but can be obtained with reasonable reliability. Effective therapies for clinical toxicity have been described.

## 1. Introduction

Mercury is a heavy metal of known toxicity, noted for inducing public health disasters in Minamata Bay, Japan [1] and in Iraq [2–4]. The clinical impact of smaller mercury exposures remains controversial. It exists in several forms: inorganic mercury, which includes metallic mercury and mercury vapor (Hg^0^) and mercurous (Hg_2_^++^) or mercuric (Hg^++^) salts; and organic mercury, which includes compounds in which mercury is bonded to a structure containing carbon atoms (methyl, ethyl, phenyl, or similar groups). The biological behavior, pharmacokinetics, and clinical significance of the various forms of mercury vary with chemical structure. There is some interconversion in vivo between the various forms of mercury. Inhaled elemental mercury vapor, for example, is easily absorbed through mucus membranes and the lung and rapidly oxidized to other forms (but not so quickly as to prevent considerable deposition of elemental mercury in the brain). Methyl mercury is easily absorbed through the gut and deposits in many tissues, but does not cross the blood-brain barrier as efficiently as elemental mercury; however, on entering the brain it is progressively demethylated to elemental mercury [5]. Mercury salts, in contrast, tend to be insoluble, relatively stable, and poorly absorbed.

Human toxicity varies with the form of mercury, the dose and the rate of exposure. The target organ for inhaled mercury vapor is primarily the brain [5]. Mercurous and mercuric salts chiefly damage the gut lining and kidney [5], while methyl mercury is widely distributed throughout the body [5]. Toxicity varies with dosage: large acute exposures to elemental mercury vapor induce severe pneumonitis, which in extreme cases can be fatal [5]. Low-grade chronic exposure to elemental or other forms of mercury induces subtler symptoms and clinical findings, as discussed hereinafter.

There is considerable controversy about the clinical significance of exposure to the various forms of mercury and some disagreement regarding techniques for clinical assessment of mercury burden. This paper is intended to review published data on these issues and to assess published clinical experience using DMPS to remove mercury from the human body. Most of the authors cited hereinafter consider DMPS to be a stronger chelator than DMSA, with one exception citing evidence that DMSA is more effective at removing organic mercury [6]. This is a complicated issue. The absorption of DMPS and DMSA by ingestion is highly variable from one patient to the next; DMPS can be given intravenously, while DMSA cannot. DMPS is considerably safer than penicillamine or British anti-Lewisite, as discussed hereinafter. It is available for compounding in the United States and is available over the counter in Germany.

## 2. Sources of Mercury Exposure

Most human exposure to mercury is caused by outgassing of mercury from dental amalgam, ingestion of contaminated fish, or occupational exposure, according to the World Health Organization [7, 8].

Mercury exists in nature primarily as elemental mercury or as a sulfide and is found in the earth's crust at approximately 0.5 parts per million. Atmospheric exposures occur from outgassing from rock or through volcanic activity. Human sources of atmospheric mercury include coal burning [9] and mining (mercury and gold in particular). Atmospheric elemental mercury settles in water, where it is converted by microorganisms into organic (methyl or ethyl) mercury, which is ingested by smaller creatures which are eventually consumed by larger fish. Fish at the top of the food chain (e.g., tuna, swordfish, or shark) may concentrate considerable mercury in their tissues.

Human mercury exposures occur chiefly [7, 8] through inhalation of elemental mercury vapor via occupational or dental amalgam exposure or through ingestion of mercury bonded to organic moieties (methyl, dimethyl, or ethyl mercury), primarily from seafood. Most human metallic mercury exposure comes from mercury vapor outgassing from amalgam fillings, at a rate of 2 to 28 micrograms per facet surface per day, of which about 80% is absorbed, according to the World Health Organization [7, 8] and Berglund et al. [10]. A less common source of mercury vapor is spilled mercury [11], and there is a report in the literature of Idiopathic Thrombocytopenic Purpura [12] caused by vacuuming spilled mercury (thereby producing a major acute exposure to mercury vapor).

Methyl and dimethyl mercury (organic mercury) usually originate from biological sources, chiefly fresh or salt water fish. Over three thousand lakes in the United States have been closed to fishing due to mercury contamination [5] and many species of ocean fish are also tainted with considerable concentrations of mercury [13].

## 3. Pharmacokinetics of Mercury Exposure

### 3.1. Inorganic Mercury

#### 3.1.1. Elemental or Metallic (Hg^0^) Mercury

Approximately 80% of metallic mercury vapor outgassed from amalgams is absorbed through inhalation [10, 14, 15], compared with about 7 to 10% absorption of ingested metallic mercury [5], and about 1% absorption of metallic mercury through skin contact [5]. On entry to the body, mercury vapor has great affinity for sulfhydryl groups and bonds to sulfur-containing amino acids throughout the body. Mercury vapor is transported to the brain [16], either dissolved in serum or adherent to red cell membranes. Metallic mercury passes easily through the blood brain barrier [17] and through the placenta, where it lodges in the fetal brain [18]. Metallic mercury is, however, rapidly oxidized to mercuric mercury on entry to the blood stream [5], although not so quickly as to prevent considerable uptake by the central nervous system while still in the metallic form.

In addition to the brain [16, 19–26], metallic mercury is also deposited in the thyroid [5, 19, 21], breast [27], myocardium [28, 29], muscles [5, 21], adrenals [5], liver [5, 30–32], kidneys [5, 7, 8, 19, 20, 23, 30–32], skin [5, 7, 8], sweat glands [5], pancreas [5], enterocytes [5, 30], lungs [5, 23, 30], salivary glands [5], testes, and prostate [5] and may be associated with dysfunction of those organs. Mercury also has affinity for binding sites on the surface of T cells and for sulfhydryl groups influencing T cell function [33, 34]. Mercury deposits readily in placenta and fetal tissues and is found in breast milk. [5, 18, 31, 35]

Metallic mercury is largely excreted as mercuric mercury [5]. The excretory half lives of metallic and mercuric mercury vary widely, depending on the organ of deposition and redox state, with values ranging from a few days to several months [5], with some pools (e.g., CNS) having a half life exceeding several years [5]. Hair mercury does not correlate with brain content of metallic mercury [5]. These complexities make accurate assessment of body burden challenging (see Section 9 hereinafter).

#### 3.1.2. Mercurous (Hg_2_^++^) Mercury

Mercurous mercury salt in the form of Hg_2_Cl_2_ (calomel) is poorly soluble in water and poorly absorbed by the intestine, although some portion is thought to undergo oxidation to more readily absorbable forms [36]. It is doubtful that mercurous mercury survives in the body, other than as a transitional form between metallic and mercuric mercury [5].

Some absorption evidently occurs, however, as calomel is occasionally associated with pink disease, or acrodynia.

#### 3.1.3. Mercuric (Hg^++^) Mercury

Historically, mercuric chloride (HgCl_2_) was used as a preservative and for development of photographic film and was ingested accidentally or as a suicide measure. It is a component of some skin-lightening creams. Only about 2% of ingested mercuric chloride is absorbed initially [37], although it is believed that its corrosive effect on the intestine may increase permeability and, hence, absorption, with prolonged exposure [38]. Available data on skin penetration of mercuric mercury are insufficient to make quantitative comparison with ingestion or with metallic mercury.

Like metallic mercury, mercuric mercury in the bloodstream adheres to sulfhydryl groups on erythrocytes, metallothionein, or glutathione or is suspended in plasma [26]. Mercuric mercury does not cross the blood-brain barrier efficiently, but it does accumulate in quantity in the placenta, fetal tissues, and amniotic fluid [35]. Evidence exists showing transport of mercuric mercury via one or more amino acid transporters [39], particularly that for cysteine, which may account for accumulation in the brain [5]. Much of the body burden of mercuric mercury resides in the proximal convoluted renal tubule [40] bonded to metallothionein [41]. Significant deposition also occurs periportally in the liver [42] and lesser amounts in epithelial tissues, choroidal plexus, and testes.

Excretion of mercuric mercury is largely through urine and stool, although significant amounts are shed through sweat, tears, breast milk, and saliva [5, 43]. Half lives appear to be multiphasic, as with metallic mercury, with human studies suggesting an effective half life of 42 days for 80% of an oral tracer dose; the other 20% did not appear to have a measureable rate of excretion [44]. This may reflect demethylation to metallic mercury in the brain and other organs or mechanisms yet to be determined.

### 3.2. Organic Mercury Compounds

Most available data on organic mercury compounds refer to methyl mercury, which is a major source of human mercury exposure, is found naturally in fish, and is relatively stable. Ethyl mercury behaves in a similar fashion to methyl mercury at the cellular level, but with an excretory half life about one third as long [5].

Methyl mercury vapor is absorbed with similar (80%) efficiency as metallic mercury vapor [5]. Intestinal absorption of methyl mercury from fish is also fairly efficient, as is absorption through the skin [5]. On entry to the bloodstream, methyl mercury adheres to sulfhydryl groups, particularly to those in cysteine. Methyl mercury is deposited throughout the body, with equilibrium between blood and body occurring approximately four days after exposure [45]. Distribution to peripheral tissues seems to occur through one or more transporters, especially the cysteine transporter, probably adherent to the sulfhydryl group in cysteine [5].

Concentration of methyl mercury occurs in the brain, liver, kidneys, placenta, and fetus, especially in the fetal brain, as well as in peripheral nerves and bone marrow [5]. Deposited methyl mercury slowly undergoes demethylation to inorganic mercury [46].

The excretory half life of methyl mercury in man is about 70 days, with approximately 90% being excreted in stool. Some degree of enterohepatic circulation apparently occurs. Perhaps 20% of methyl mercury is excreted in breast milk, with the actual amount varying with severity of exposure [5]. Hair mercury reflects blood methyl mercury at the time of incorporation, but not elemental mercury [47], and hence is not a good index of total body burden [5], given the short half life of methyl mercury in blood.

Dimethyl mercury is also efficiently absorbed through the skin, and there is a reported death of a scientist caused by minimal skin contact [48].

## 4. Toxicity

### 4.1. Inorganic Mercury

#### 4.1.1. Metallic Mercury Vapor

Mercury in all forms poisons cellular function by altering the tertiary and quaternary structure of proteins and by binding with sulfhydryl and selenohydryl groups. Consequently, mercury can potentially impair function of any organ, or any subcellular structure. The chief target organ of mercury vapor is the brain, but peripheral nerve function, renal function, immune function, endocrine and muscle function, and several types of dermatitis have been described [49].

With massive acute exposure to mercury vapor, erosive bronchitis and bronchiolitis potentially leading to respiratory failure may be accompanied by CNS symptoms such as tremor or erethism [50].

Chronic exposure to clinically significant doses of mercury vapor usually produces neurological dysfunction. At low-level exposures, nonspecific symptoms like weakness, fatigue, anorexia, weight loss, and gastrointestinal disturbance have been described [51]. Higher exposure levels are associated with mercurial tremor: fine muscle fasciculations punctuated every few minutes by coarse shaking. Erethism may also be observed: severe behavior and personality changes, emotional excitability, loss of memory, insomnia, depression, fatigue, and in severe cases delirium and hallucination [10]. Gingivitis and copious salivation have been described [5].

These symptoms may regress with cessation of exposure, but in many cases do not. Persistent neurological symptoms are common [52].

#### 4.1.2. Mercurous Mercury

Calomel (Hg_2_Cl_2_) is still used in some regions of the world as a laxative. Although poorly absorbed, some is converted to mercuric mercury, which is absorbed, and induces toxicity as expected with mercuric mercury.

#### 4.1.3. Mercuric Mercury

Acute poisoning with mercuric salts (typically HgCl_2_) generally targets the gastrointestinal tract and the kidneys. Extensive precipitation of enterocyte proteins occurs, with abdominal pain, vomiting, and bloody diarrhea with potential necrosis of the gut mucosa. This may produce death either from peritonitis or from septic or hypovolemic shock. Surviving patients commonly develop renal tubular necrosis with anuria [53].

Chronic poisoning with mercury salts is rare, usually also involving concomitant occupational exposure to mercury vapor. Kidney toxicity involves either renal tubular necrosis or autoimmune glomerulonephritis, or both [53]. Immune dysfunctions include hypersensitivity reactions to mercury exposure, including asthma and dermatitis, various types of autoimmunity [54], and suppression of natural killer cells [55] and disruption of various other lymphocyte subpopulations [5].

Brain dysfunction is less evident than with other forms of mercury. Thyroid dysfunction seems associated with inhibition of the 5′ deiodonases, with decreased free T3 and increased reverse T3 [56]. Accumulation in the testicles appears to inhibit spermatogenesis [57]. Atrophy and capillary damage have been described in thigh muscle [58].

### 4.2. Organic Mercury

Methyl mercury reacts with sulfhydryl groups throughout the body, therefore potentially interfering with the function of any cellular or subcellular structure. Mercury is believed to interfere with DNA transcription and protein synthesis [59], including protein synthesis in the developing brain, with destruction of endoplasmic reticulum and disappearance of ribosomes [60]. Evidence suggests disruption of numerous subcellular elements in the central nervous system and other organs and in mitochondria; adverse effects have also been described on heme synthesis [61], cell membrane integrity in many locations [5], free radical generation [27, 62, 63], neurotransmitter disruption, and stimulation of neural excitoxins [5], resulting in damage to many parts of the brain and peripheral nervous system [5].

Methyl mercury has been associated with reduction in Natural Killer cell activity [64–67], as well as an imbalance in Th2 : Th1 ratios favoring autoimmunity [34, 68, 69]. Mercury is also possibly associated with disruption of DNA repair [5, 27]. The affinity of mercury for sulfhydryl groups of the mitochondrial oxidative phosphorylation complex [70] associated with destruction of mitochondrial membranes may contribute to chronic fatigue syndrome.

## 5. Clinical Presentation

### 5.1. Inorganic

#### 5.1.1. Elemental (Metallic) Mercury

Acute exposure to a large quantity of mercury vapor induces pneumonitis, as discussed previously. Symptoms of low-grade chronic exposure are more subtle and nonspecific: weakness, fatigue, anorexia, weight loss, and gastrointestinal distress [5], sometimes referred to as micromercurialism [71]. At higher exposures, the mercurial fine tremor punctuated by coarse shaking occurs; erethism, gingivitis, and excessive salivation have also been described [5], as has immune dysfunction [34].

Objective findings include altered evoked potentials and decreased peripheral nerve conduction velocity [72]. Objective measures of short-term memory may be inversely correlated with urinary mercury in chloralkali workers [73]. Reduced color vision and visual acuity have also been observed [74]. Changes in coordination, tremor, mental concentration capacity, facial expression, and emotional state are also described [75], as are polyarthritis, various forms of dermatitis, and a syndrome mimicking pheochromocytoma [76].

Subtler clinical findings among dentists have been documented, including delayed reaction time, poor fine motor control, and deficits in mental concentration, vocabulary, task switching, and the One Hole test, as well as mood lability, all correlating with urinary mercury excretion [75]. Evidence also links elemental mercury to depression, excessive anger, and anxiety [77], as well as acute myocardial infarction, lipid peroxidation, and carotid atherosclerosis, in Finland [78]; the Finnish experience may possibly be explained by dietary selenium deficiency, since selenium antagonizes mercury toxicity. Other investigators, however, have described associations between mercury and hypertension, lipid peroxidation, ischemic heart disease, and stroke [79].

#### 5.1.2. Mercuric Salts

Ingestion of mercuric chloride produces extensive precipitation of intestinal mucosal proteins, mucosal necrosis, generalized abdominal pain, bloody diarrhea, and shock. If the patient survives, acute renal failure may follow [5].

### 5.2. Organic Mercury

Methyl mercury and ethyl mercury produce similar signs and symptoms. Most published data refer to methyl mercury. Symptoms relate more to magnitude of methyl mercury retention than to the rate of deposition. Acute exposures tend to have a latency period of one or more weeks; once acquired, toxic doses are cleared slowly, if at all [5].

Massive prenatal poisoning may induce a form of cerebral palsy [5]. Lesser prenatal doses have been associated with neurodevelopmental delays and cognitive deficits [80–82].

Postnatal exposures generate a range of symptoms ranging from paresthesias, with lesser exposures, to ataxia, visual, auditory, and extrapyramidal impairments with moderate exposures and clonic seizures in more severe exposures, as in Minamata [1] and Iraq [2–4].

Objective physical findings are similar to those seen with elemental mercury exposure.

## 6. Laboratory Assessment of Mercury Exposure

Given the wide range of excretory half lives of the various mercury pools, discussion of laboratory assessment will combine the various forms into one discussion. It is important to recall that blood, hair, and urine mercury levels reflect recent exposure and do not correlate with total body burden [83–86]. Blood and urine levels correlate fairly well to each other, but not to total body burden [87]. With half life of all mercury pools in the blood estimated to be in the range of three to five days [88], during which either excretion or deposition in solid organs occurs, more accurate means of estimating body burden have been required.

That being said, the US federal biological exposure index (BEI) is currently set at 50 mcg/L urine. Aside from the obvious problems associated with basing a monitoring index on a measurement which only reflects current or recent exposure, and not overall body burden, several clinical studies show objective symptoms well below 50 mcg/L, with many proband values extending down into the low end of the reference range for urinary mercury excretion [75, 89–94], effectively rendering the US federal BEI useless for clinical or investigational purposes. Similar criticisms have been made of the EPA Reference Dose for methylmercury [95]. As summarized by Kazantzis, “it has not been possible to set a level for mercury in blood or urine below which mercury related symptoms will not occur” [96].

Because of these difficulties, provocation with a chelator has been proposed as providing a more reliable estimate of body burden, and DMPS (2,3 Dimercapto-1-Propanesulfonate) has been found by a number of investigators to provide a reliable estimate of body burden, safer than British Anti-Lewisite and more potent than DMSA [75, 97–101].

## 7. DMPS: Safety

DMPS is an analog of British Anti-Lewisite (BAL) with high affinity for mercury. Due to its superior safety, it has been widely used in Germany for the past fifty years and is available over the counter in that country. Protocols determining the pharmacokinetics of DMPS and evaluating its use for diagnostic purposes have been published in Germany [101], Sweden [102, 103], New Zealand [100], and Mexico [104] and in the United States [105–109].

Maiorino et al. [106] gave his volunteers DMPS 300 mg orally; over 90% of the absorbed DMPS was converted rapidly to disulfide forms. Published absorption of ingested DMPS varies from 39% [107] to 60% [110]. The excretory half life of unaltered DMPS was 4.4 ± 1.1 hours. The excretory half life of the disulfide forms of DMPS was  9.9 ± 1.6  hours.

Hurlbut et al.'s [107] volunteers were given an unusually large dose of DMPS (3 mg/kg intravenously over 5 minutes). Two subjects had a transient 20 mmHg drop in systolic blood pressure during infusion, without other changes in vital signs. Excretory half life of unaltered DMPS ranged from 1.3 to 4.0 hours. Half life of the altered DMPS was from 19.8 to 37.5 hours.

In each of the cited studies, mercury output following provocation with DMPS correlated significantly with amalgam number and/or occupational or dietary exposure. There were no significant complications in any of the trials. Consequently, all the investigators but one [111] concluded that urine output provoked by DMPS represented a fair estimate of body burden.

## 8. DMPS: Efficacy

Each of the test trials cited in the previous section and others [112] showed statistically significant increases in urinary mercury output with administration of DMPS. With prolonged treatment, evidence of decreased body burden has been inferred [113].

Several controlled clinical trials support this conclusion. The largest was undertaken in the Phillippines in a gold mining area [114]. Workers in gold mining who sustained ongoing exposure to elemental mercury were compared to people living downstream who ate fish, which contained considerable methyl mercury, and to controls without significant known mercury exposure. Probands from the two exposed areas were chosen with elevated blood, urine and hair mercury levels, and appropriate symptoms (tremor, sleeplessness, memory loss, etc.)[115]; controls had normal levels and were asymptomatic.

One hundred six probands completed the fourteen-day trial with oral DMPS 400 mg per day. The only complication was an allergic rash in one patient, who was excluded from the trial. Blood mercury did not decrease during the trial, despite increases in urine mercury up to 85-fold.

Despite the short (fourteen-day) duration of the trial, significant improvements were observed in objective measures like hypomimia, Romberg test, tests for tremor and ataxia, pencil tapping, and Frostig visual perception. Most of the patients reported subjective improvement in memory, sleeplessness, metallic taste, fatigue, anxiety, and paresthesias. Treatment efficacy was similar in the metallic mercury group (miners) and in the methyl mercury group (downstream fish eaters). Similar results were presented in a parallel study by Drasch et al. [115].

A university case report from the United States of treatment of occupational exposures to mercury vapor [116] showed relief of muscle twitching, arthralgias, paresthesias, night sweats, weight loss, and excessive salivation following two weeks of oral DMPS 100 mg TID followed by DMPS 100 mg QID for an additional six weeks. Reduction of symptoms closely paralleled urine mercury output, which tapered over time.

## 9. Discussion

Mercury toxicity is not often included in the differential diagnosis of common subjective complaints such as fatigue, anxiety, depression, odd paresthesias, weight loss, memory loss, and difficulty concentrating, but these are the symptoms of low-grade chronic mercury exposure described by the investigators cited previously. Given the ability of the various forms of mercury to deposit in most parts of the human body, the range of symptoms potentially caused by mercury is quite large.

Animal studies linking mercury toxicity to neurodegenerative diseases [117, 118] raise clinical concern, as do a series of associations between mercury and neurodegenerative diseases in humans [119–123].

Mercury exposure is not insignificant according to WHO, as cited previously, and the NHANES reports suggest widespread exposure in the United States, especially among women [124, 125].

Diagnosis of mercury overload is difficult. The commonly used modalities (blood, urine, and/or hair levels) do not correlate with total body burden and offer little diagnostically useful information. Provocation with DMPS appears to offer a more accurate assessment of body burden.

Since provocation is safe and inexpensive, indications for provocation must rest on clinical grounds: does the patient have multiple, vague symptoms similar to those described in the mercury literature, without other plausible, and potentially reversible, explanation? Is there a significant history of mercury exposure: multiple amalgam fillings, high seafood intake, and history of multiple thimerosal-containing vaccinations or significant occupational exposures? Is there a family history of Alzheimer's, Parkinson's, or other diseases with postulated links to mercury exposure? Is there a history of known glutathione transferase (GST) polymorphisms, which decrease the body's ability to clear heavy metals like mercury?

If so, then provocation with a chelator may be indicated. Published protocols [126–130] exist which call for provocation with DMPS with or without EDTA, in sequence. These are designed for safety, and for diagnostic breadth. DMPS has far better affinity for mercury than EDTA, but EDTA is more effective in removing lead, cadmium, nickel, and other toxic metals. Provocation with both gives a fuller picture of overall metal burden. Patients with GST enzyme abnormalities may also receive glutathione to expedite excretion of chelated metal. For unknown reasons, patients with GST polymorphisms tend to excrete mercury later in their course of treatment than other heavy metals [131]; this can sometimes produce early false negatives for mercury, due to preferential excretion of lead and other metals. All effective chelation protocols call for replacement of beneficial minerals, which are also removed by EDTA and DMPS.

There are currently no consensus criteria for the diagnosis of mercury overload, nor for overload of other toxic metals. Clinicians who specialize in this area generally consider a provoked urine metal output more than 2 standard deviations above the NHANES reference range a positive result.

Further research is required to clarify the relation between provoked urine results and clinical disease and to document clinical outcomes.
